# Failure to initiate trauma team activation for patients who meet the criteria in a level 1 paediatric trauma centre: which patients are missing out?

**DOI:** 10.1111/ans.17906

**Published:** 2022-07-14

**Authors:** Jonghoo Sung, Angela Yao, Georgia Antoniou, Rebecca Cooksey, Jacqueline Winters, Michael Ee, Nicole Williams

**Affiliations:** ^1^ Faculty of Health and Medical Sciences The University of Adelaide Adelaide South Australia Australia; ^2^ Department of Orthopaedic Surgery Women's and Children's Hospital Adelaide South Australia Australia; ^3^ Paediatric Major Trauma Service, Division of Surgical Services Women's and Children's Hospital Adelaide South Australia Australia; ^4^ Department of Paediatric Surgery Women's and Children's Hospital Adelaide South Australia Australia; ^5^ Department of Paediatric Medicine Women's and Children's Hospital Adelaide South Australia Australia

**Keywords:** emergency medical services, orthopaedic surgery, paediatric hospitals, paediatric surgery, trauma centres, triage, trauma

## Abstract

**Background:**

Trauma team activation (TTA) is paramount in the early hospital management of trauma patients. This study aimed to evaluate factors which contribute to failure to activate the Trauma team for management of paediatric trauma.

**Methods:**

A retrospective cohort study of Emergency Department (ED) presentations at the paediatric major trauma hospital in Adelaide, South Australia was conducted over a 16‐month period. Data from the hospital's trauma registry, individual case files and digital medical records were evaluated to determine factors that were associated with no TTA.

**Results:**

During the study period, 617 trauma patients who met Level 1 or Level 2 TTA criteria attended the trauma centre. For 29 (4.7%) of these patients, there was no TTA. Predictors of no TTA included sustaining abdomen and/or pelvis injuries compared to limb injuries (unadjusted odds ratio [OR] = 10.59, 95% confidence interval [CI] 1.98–56.69, *P* = 0.006), sustaining non‐accidental injury (NAI) versus an injury with vehicle involvement (OR = 30.13, 95% CI 6.43–141.21, *P* < 0.001), and arriving via emergency medical retrieval service compared to private vehicle (OR = 14.23, 95% CI 3.94–51.36, *P* < 0.001). No patients transferred directly to Paediatric Intensive Care Unit (PICU), or High Dependency Unit (HDU) received an appropriate TTA.

**Conclusion:**

Multiple factors were associated with no TTA in paediatric trauma patients. The results highlight that even in PICU and HDU admissions and transfer patients, vigilant clarification of mechanism of injury and potential for occult injuries should be undertaken to ensure appropriate TTA and improve patient outcome.

## Graphical abstract

Evaluating factors which contribute to failure to activate the Trauma Team for management of paediatric trauma is paramount in optimising patient care. The results highlight that even in Paediatric Intensive Care Unit and High Dependency Unit admissions and transfer patients, vigilant clarification of mechanism of injury and potential for occult injuries should be undertaken to ensure appropriate TTA and improve patient outcome.

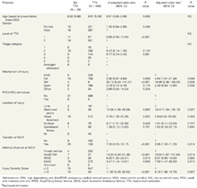

## Introduction

Trauma teams are a multidisciplinary group of healthcare professionals that provide prompt assessment, treatment and resuscitation for injured patients.^1^ An estimated 10–15% of paediatric trauma patients have life‐threatening injuries that require efficient and rapid management.^2^ The American Academy of Paediatrics and American College of Surgeons (ACS) recommend early trauma team activation (TTA) to provide optimal care in severely injured children^3^ and it is important for paediatric trauma patients to be triaged appropriately on arrival to the Emergency Department (ED) to receive adequate level of care.^4^ Paediatric specific criteria have been developed but there is no perfect triage or TTA system and overtriage or undertriage cannot be completely avoided.^5^

Overtriage may result in wastage of resources including personnel, time and equipment,^6^ whereas undertriage may result in potential harm^2^ as well as delay to assessment, investigation and treatment. Mortality in paediatric trauma patients was shown to be doubled in hospitals with lower trauma triage rates.^7^ Undertriaged adult patients are more likely to have increased hospital length of stay, suffer at least one complication and have increased mortality.^8^ Therefore, the priority is often to reduce undertriage and ensure subspecialist input to avoid preventable morbidity and mortality,^9^ while acknowledging that this may conversely increase overtriage for those with less severe or negligible injuries.^5^, ^9^

This study was initiated following a coronial inquest^10^ into the death of a nine‐year‐old girl who did not receive TTA at our institution, despite meeting hospital criteria for Level 2 TTA based on mechanism of injury, with observations in the department meeting criteria for upgrade to Level 1 TTA. The coroner determined the death was due to undiagnosed perforated bowel and peritonitis as a result of a fall. Had this patient received the appropriate Level 1 TTA, a surgical registrar would have assessed her in the ED. This may have facilitated identification of the intra‐abdominal injury.

The aim of this study was to identify factors leading to failure of TTA in order to develop strategies to minimize this occurrence.

## Methods

The study setting was the Women's and Children's Hospital (WCH), Adelaide, Australia, the paediatric major trauma centre for patients up to their 16th birthday from South Australia, Northern Territory, and adjacent parts of Western Victoria and South‐West New South Wales. Following hospital Human Research Ethics Committee approval (Audit 839A), an observational, retrospective cohort study was conducted to include patients who had presented over a 16‐month period.

The WCH uses a two‐tier system of TTA and TTA can be performed by any staff member on recognition that the patient meets the criteria of the Paediatric South Australian Trauma System TTA Criteria Version 2.1—July 2014 (Tables 1, 4). The staff member contacts the hospital switchboard to advise the age, expected time of presentation or current location within the hospital and TTA Level (1 or 2). Members of the TTA response (Table 2) are notified via pager or mobile phone text message. TTA is commonly performed by a senior ED clinician following receipt of a Government Radio Network (GRN) call to advise of an incoming patient or by the duty triage nurse for patients arriving by private transport. In our institution, ED physicians are Fellows of Australasian College for Emergency Medicine (FACEM), or Fellows of the Royal Australasian College of Physicians (FRACP), with majority having completed trauma courses such as Early Management of Severe Trauma (EMST).

**Table 1:** Triage criteria for level 1 trauma team activation and level 2 trauma team activation

Level 1 Trauma Team Activation:
Physiological Profile (Worst pre‐hospital or on arrival status):
Abnormal respiratory function
Compromise of airway and/or breathing
Laboured Respirations
Cyanosis
Low saturations (≤90%)
Abnormal heart rate and blood pressure
GCS <13
Injury Profile:
Airway compromise (including intubation or attempted intubation)
Flail chest or subcutaneous emphysema
Ongoing uncontrolled significant haemorrhage
Abdomen—severe pain, distension and/or involuntary guarding
Penetrating injury to head, neck or torso (including upper arm, upper leg and perineum)
Severe maxillofacial injury
Major pelvic fractures
Spinal injury with neurological signs
Femur fracture plus one other long bone fracture
Amputation or severe crush, proximal to the wrist or ankle or ischaemic limb/s
Burns >20% BSA and/or inhalation burns
MedSTAR primary trauma retrieval (retrieval direct from incident)
Level 2 Trauma Team Activation:
MVC ≥60 km/h or vehicle severely damaged
Ejection from a vehicle or death of an occupant
Pedestrian or cyclist (pedal or motor) struck (or fall from) at ≥30 km/h
Prolonged extrication time (≥30 min)
Fall >2 m (age < 2 years—fall from >2 times the child's body height)
Fall from or kicked by a horse
Handlebar (or other significant blunt injury) to the abdomen
Hanging/traumatic asphyxiation
Other significant mechanism
MedSTAR secondary trauma retrieval (inter‐facility transfer, except for those who meet Level 1 criteria)

Abbreviations: BSA, body surface area; GCS, Glasgow coma score; MedSTAR, emergency medical retrieval service; MVC, motor vehicle collision.

**Table 2:** Team members notified with a level 1 and level 2 trauma team activation

Level 1	Level 2
Senior PED staff medical practitioner (consultant or senior registrar) Surgical Registrar	Senior PED staff medical practitioner (consultant or senior registrar)
Orthopaedic Registrar	PED Registrar
PICU Registrar	PED Nurses
PED Registrar	Trauma Nurse (in hours only)
Anaesthetic Registrar (after hours only)	Radiology (as requested)
Medical Registrar (after hours only)	PED patient service attendant
PED Nurse	Social Worker (in hours) or after hours nursing management facilitator (after hours)
PICU Nurse	
Trauma Nurse (in hours only)	
Radiographer	
PED Patient Service Attendant	
Social Worker (in hours) or after hours nursing management facilitator (after hours)	

Abbreviations: ED, emergency department; PED, paediatric ED.

The WCH Trauma Nurse Consultant enters data into the WCH Trauma Database for reporting to the Australian Trauma Registry for all patients seen at WCH who meet TTA criteria. Patients who meet criteria but did not receive TTA are identified by Monday–Friday Trauma Nurse Consultant review of the Emergency Department Information System (EDIS). These patients are also entered into the Trauma Database. This study included all patients who presented to WCH over the 16‐month period and met TTA criteria. Additional information for the audit was sourced from patient case files, monthly Trauma Audit minutes and the statewide Open Architecture Clinical Information Systems (OACIS) database.

Information collected included age, gender, TTA level (i.e., Level 1, 2 or not activated), ED triage level, mechanism of injury, criteria met for TTA, whether the patient was a direct admission to Paediatric Intensive Care Unit (PICU) or High Dependency Unit (HDU), anatomic location of injury, inter‐hospital transfers, method of arrival to WCH and Injury Severity Score (ISS).

Mechanism of injury was classified as motor vehicle accident (MVA), fall (greater than 2 m, or from double the child's height if aged <2 years), sporting injury, non‐accidental‐injury (NAI) or other.

Data analysis was performed with statistical software IBM SPSS Version 19. Chi‐square analysis was used to determine the association between TTA status and each of the possible categorical predictor variables. Logistic regression analysis was used to determine significant predictors of no TTA. Variables with *P* < 0.1 at the univariable level were then included in a multivariable logistic regression model using backward elimination (conditional) with probability for removal set at *P* > 0.1 and probability for entry set at *P* < 0.05. A *P* < 0.05 was used for statistical significance.

## Results

**Fig. 1:**
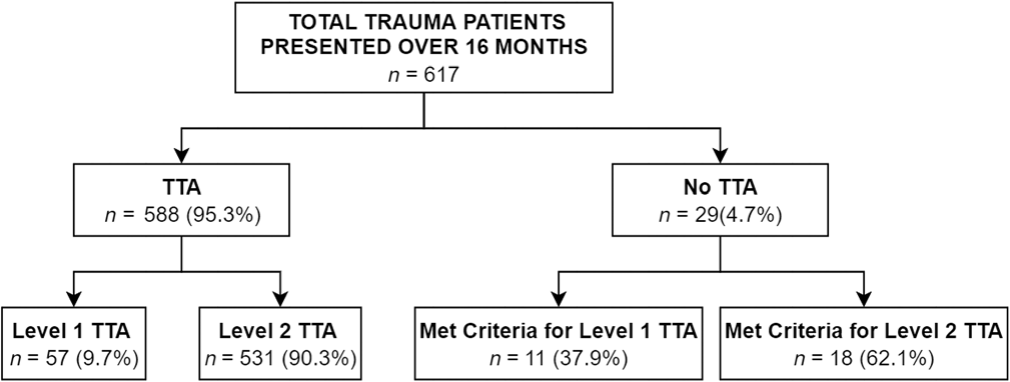
Flowsheet demonstrating the number of patients each classified into the appropriate levels of TTA when activated and not activated.

Six‐hundred and seventeen trauma patients were seen at WCH during the study period; 29 patients who had met TTA criteria had failure of TTA (Fig. 1), an incidence of 4.7% over the 16‐month study period. Factors associated with failure of TTA are presented in Table 3.

**Table 3:** Univariable and multivariable logistic regression analysis of risk factors associated with no trauma team activation

Predictor		No TTA (*N* = 29)	TTA (*N* = 588)	Unadjusted odds ratio (95% CI)	*P*‐value	Adjusted odds ratio^†^ (95% CI)	*P*‐value
Age (years) at presentation (mean(SD))		6.82 (5.88)	9.61 (5.38)	0.91 (0.85–0.98)	0.008		NS
Gender							
	Female	13	221	1.35 (0.64–2.86)	0.434		
	Male	16	367	1			
Level of TTA							NS
	1	11	57	5.69 (2.56–12.65)	<0.001		
	2	18	531	1			
Triage category							NS
	1	5	70	1			
	2	10	336	0.42 (0.14–1.26)	0.120		
	3	8	145	0.77 (0.24–2.45)	0.661		
	4	0	35	–			
	5	0	2	–			
	Arranged admission	6	0	–			
Mechanism of injury							
	MVA	5	226	1		1	
	Fall	10	156	2.90 (0.97–8.64)	0.056	4.64 (1.01–21.28)	0.048
	NAI	4	6	30.13 (6.43–141.21)	<0.001	19.90 (2.36–168.05)	0.006
	Sport	8	166	2.18 (0.70–6.78)	0.179	2.40 (0.53–10.90)	0.258
	Other	2	34	2.66 (0.50–14.25)	0.254		
PICU/HDU admission							
	No	21	588	1		1	
	Yes	8	0	–		–	
Location of injury							
	Limb	2	161	1		1	
	Abdomen/pelvis	5	38	10.59 (1.98–56.69)	0.006	13.47 (1.34–135.15)	0.027
	Head/face/neck	15	212	5.70 (1.28–25.26)	0.022	2.94(0.33–25.92)	0.333
	Multiple	6	79	6.11 (1.21–30.98)	0.029	1.61 (0.11–23.03)	0.727
	Spine/back/thorax	1	58	1.39 (0.12–15.60)	0.791	1.83 (0.10–32.13)	0.680
	Nil	0	40	–		‐	
Transfer to WCH							
	No	9	450	1		1	
	Yes	20	138	7.20 (3.20–16.17)	<0.001	3.85 (1.32–11.25)	0.014
Method of arrival at WCH							
	Private vehicle	4	252	1		1	
	MedSTAR	7	31	14.23 (3.94–51.36)	<0.001	13.04 (1.67–101.84)	0.014
	RFDS	3	30	6.30 (1.35–29.51)	0.019	0.97 (0.05–18.40)	0.985
	SAAS	15	272	3.47 (1.14–10.61)	0.029	7.50 (1.47–38.20)	0.015
	Unknown	0	3	–		–	
Injury Severity Score	≤12	15	547	1		1	
	>12	13	40	11.85 (5.28–26.62)	<0.001	5.10 (1.56–16.70)	0.007

Abbreviations: HDU, high dependency unit; MedSTAR, emergency medical retrieval service; MVA, motor vehicle accident; NAI, non‐accidental injury; PICU, paediatric intensive care unit; RFDS, Royal Flying Doctor Service; SAAS, South Australian Ambulance Service; TTA, trauma team activation.

^**†**^ Backward elimination.

### Age

The mean age at presentation of patients with failure of TTA was 6.82 years compared to 9.61 years for patients who received TTA. The odds of failure of TTA decreased by a multiplicative factor of 0.91 (95% CI 0.85–0.98, *P* = 0.008) for every 1‐year increase in age.

### Gender

There was no association between gender and TTA status with 13/234 (5.6%) female and 16/383 (4.2%) male patients not receiving TTA (*P* = 0.44).

### Level of trauma

There was no TTA for 11/68 (16.2%) patients who met Level 1 criteria on presentation and 18/549 (3.3%) patients who met Level 2 criteria. Included in the 549 patients who met criteria for Level 2 TTA on presentation were eight who were upgraded to Level 1 on further assessment, one of these had no TTA. The odds of no TTA were higher in patients meeting Level 1 TTA criteria compared to patients meeting Level 2 TTA criteria (OR = 5.69, 95% CI 2.56–12.65, *P* < 0.001). The categorisation of level of trauma of the study patients is summarized in Figure 1.

### Mechanism

The most common mechanism of injury was motor vehicle accident (MVA), involving 231 (37.4%) patients with five (2.2%) not receiving TTA. Compared with MVA, non‐accidental injury (NAI) was associated with increased risk of no TTA (OR = 30.13, 95% CI 6.43–141.21, *P* < 0.001). The odds of no TTA for injuries sustained through a fall approached significance (OR = 2.90, 95% CI 0.97–8.64, *P* < 0.056).

### PICU/HDU

8/617 (1.3%) of trauma patients were admitted directly to PICU/HDU, bypassing the ED. All eight patients did not have TTA. 21/609 (3.4%) of patients who did not require PICU/HDU admission did not have TTA (*P* < 0.001). Monthly Trauma Audit minutes were reviewed to determine if any deficiencies in trauma care were identified for these patients (Table 4).

**Table 4:** Deficiencies in care identified at monthly trauma audit for cases admitted to Paediatric intensive care unit without trauma team activation

Case	Mechanism of Injury	Location of injury	Deficiencies in care identified at Trauma Audit
1	Fall	Head/face/neck (intracranial bleed)	Nil
2	Fall	Head/face/neck (intracranial bleed)	Nil
3	Other (burns)	Multiple	Burns pathway not adhered to: no NGT, no IDC, no bloods collected, unsatisfactory wound care
4	Other (burns)	Multiple	Nil
5	MVA	Lower limb	Inappropriately taken by SAAS to adult MTC then transferred to WCH PICU, medication dosing error at adult MTC
6	Fall	Head/neck/face (intracranial bleed)	May have been intubated earlier in PED
7	MVA	Lower limb	Inappropriately taken by SAAS to adult MTC then transferred to WCH PICU. Incorrect fluid dosage at adult MTC. Femoral nerve block performed but not documented.
8	Other (self‐harm)	Head/neck/face	Nil

Abbreviations: IDC, indwelling catheter; NGT, nasogastric tube; MTC, major trauma centre; PED, Paediatric Emergency Department; PICU, paediatric intensive care unit; SAAS, South Australian Ambulance Service; WCH, Women's and Children's Hospital.

### Location of injury

Compared to limb injuries, there was an increased risk of no TTA for abdomen/pelvis injuries (OR = 10.59, 95% CI 1.98–56.69, *P* = 0.006), head/face/neck injuries (OR = 5.70, 95% CI 1.28–25.26, *P* = 0.022) and multiple injuries (OR = 6.11, 95% CI 1.21–30.98, *P* = 0.029).

### Transfer patients

A 20/158 (12.7%) of patients transferred to WCH from other hospitals did not have TTA compared to 9/459 (2%) that were not transfers (OR = 7.20, 95% CI 3.20–16.17, *P* < 0.001).

### Triage category

A triage category, as distinct to the TTA system is assigned to all ED patients by the triage nurse. ED triage category did not predict failure of TTA However, six patients who had an ‘arranged admission’ to WCH PICU and were not given a triage category, did not have TTA.

### Method of arrival to the WCH

Most patients arrived at WCH via private vehicle (41.5%) or by the South Australian Ambulance Service (SAAS) (46.5%). No TTA was more likely for patients arriving by emergency medical retrieval service (MedSTAR) (OR = 14.23, 95% CI 3.94–51.36, *P* < 0.001); Royal Flying Doctors Services (RFDS) (OR = 6.30, 95% CI 1.35–29.51, *P* = 0.019); and SAAS (OR = 3.47, 95% CI 1.14–10.61, *P* = 0.029) compared with arriving by private vehicle.

### Injury severity score

Of the 615 patients that had an ISS score recorded, 562 (91.4%) had a score less than or equal to 12 and 53 (8.6%) had a score greater than 12. 15 (2.7%) of those with a score ≤12 had no TTA compared to 13 (24.5%) of those with a score >12. The odds of no TTA were significantly increased for those with an ISS score >12 (OR = 11.85, 95% CI 5.28–26.62, *P* < 0.001).

### Multivariable model

Following multivariable logistic regression analysis, the following were identified as independent predictors of no TTA: transfer to WCH versus no transfer (OR = 3.85, 95% CI 1.32–11.25, *P* = 0.014); MedSTAR transport (OR = 13.04, 95% CI 1.67–101.84, *P* = 0.014) and SAAS transport (OR = 7.50, 95% CI 1.47–38.20, *P* = 0.015) versus arrival via private vehicle; mechanism of injury NAI (OR = 19.90, 95% CI 2.36–168.05, *P* = 0.006) or fall (OR = 4.64, 95% CI 1.01–21.28, *P* = 0.048) versus MVA; location of injury abdominal/pelvic *vs*. limb (OR 13.47, 95% CI 1.34–135.15, *P* = 0.027); ISS score > 12 versus ISS score ≤ 12 (OR = 5.10, 95% CI 1.56–16.70, *P* = 0.007). All cases that were admitted to PICU/HDU did not have a TTA.

## Discussion

Our institution uses a two‐tiered trauma activation protocol, with this system shown to be effective within the context of an Australian Metropolitan major trauma hospital.^11^ TTA is known to improve acute trauma care in the ED,^12^ and it is paramount that TTA occurs when indicated. The incidence of failure to initiate TTA in our institution for patients meeting criteria during our study period was 4.7%. An undertriage rate of <5% is considered acceptable according to the American College of Surgeons‐Committee on Trauma (ACS‐COT)^13^ and the incidence of undertriage in paediatric trauma patients reported in the literature ranges from 3% to 45.8%.^11^, ^14^, ^15^, ^16^ ACS‐COT states that an overtriage rate of 30%–50% is necessary to maintain an undertriage rate of less than 10%^13^ and identifying factors that lead to undertriage may simultaneously avoid wastage of resources by overtriaging.^6^

Direct admission to PICU/HDU and transfer from another hospital were major independent predictors of failure to initiate TTA in our institution. Guidelines endorsed by the South Australia State Trauma Committee advise that upon arrival at WCH, all transferred patients meeting TTA criteria (Table 1) must have a trauma page initiated irrespective of whether the patient was initially assessed at another hospital and irrespective of whether the patient is initially assessed in WCH ED or elsewhere in the hospital. Although there is no strict timeframe a TTA must be activated within, it is recommended that a TTA is initiated in a timely manner.^17^ Although PICU/HDU services stabilize and provide supportive care for the critically ill, failure to initiate TTA may delay assessment and treatment by specialist teams, including surgical intervention and specialist radiological investigations, particularly if relevant staff are on‐call, rather than on‐site. In our institution, although an Intensivist is allocated to every patient in PICU, the patients are admitted under the initial admitting team. However, all trauma cases should be admitted under the Paediatric Surgery team as a major trauma case. If admitted under a specialty team, important aspects of standard trauma team care such as secondary and tertiary surveys may be missed, leading to unidentified injuries. Independent comprehensive assessment upon presentation is important, despite any previous assessments as the clinical picture may have changed and resources in the transferring institution may have been limited. For trauma cases admitted directly to PICU without TTA in our hospital, deficiencies in care were identified at our monthly Trauma Audits, including failure to follow our hospital pathway for burns management.

Younger patients were at increased risk of failure to initiate TTA. The most common age group for injury in Australian children is 11–16 years, followed by 1–5 years.^18^ With increasing age, the place of injury changes from home to the open roads and playgrounds,^19^, ^20^ with a corresponding increase in surgical treatment.^21^ Such factors may cause an unconscious age bias which heighten the clinicians' concerns to pre‐emptively activate a trauma call. Additionally, NAI was less likely to have a TTA compared to more obvious mechanisms such as MVA. Clinicians must always remain vigilant to the possibility of NAI and incongruous history and injuries.

Consistent with previous studies^22^, ^23^ limb injuries were more likely to receive TTA than head, neck and facial injuries and abdomen/pelvis and multiple injuries. Limb injuries are more visible than internal injuries, hence are associated with a lower risk of undertriage.^23^ This highlights the importance of comprehensive secondary survey and screening of injury profile based on mechanism of injury.

Our study showed a high number (41.5%) of self‐presentation to the ED via private vehicle. In South Australia, long ambulance waiting time has been heavily portrayed by the media.^24^, ^25^ Patient families who are aware of ambulance ramping may prefer private transportation to ED. Furthermore, Medicare does not subsidize the cost of ambulance call out fee if the patient is not covered by South Australia Ambulance Service (SAAS) Ambulance Cover. Many private insurance policies may only cover the cost of an ambulance in what they define as an ‘emergency’, and in many cases the insurers limit claims to one ambulance use per year.^26^ The lack of coverage of ambulance services may contribute to the higher number of private presentations to ED as described by the Australian Institute of Health and Welfare.^26^

Arrival to WCH via emergency retrieval service, such as MedSTAR, SAAS, and RFDS showed significantly higher odds of no TTA compared to arrival by private vehicle. The majority (87.5%) of direct PICU admissions were retrieved via these services, which played a factor in undertriage of retrieved patients. MedSTAR and RFDS provide retrieval services mostly from rural areas.^27^, ^28^ Rural areas have been extensively studied to be associated with a greater risk of undertriage,^14^, ^29^ and a lack of paediatric trauma centres, experts and resources.^14^ Failure of TTA for transferred patients may also reflect a bystander effect whereby increasing the number of healthcare professionals involved in the care of a hospitalized patient may result in decay of coordination of care. Clinicians may assume a passive role and conclude that another physician will bear the burden of authority,^30^ with failure to activate a trauma call. While rapid assessment and transfer are important, clinicians must not undervalue the role of TTA for patients arriving in ED or PICU/HDU.

Inappropriate dosing (medication and fluids) was identified for two patients taken initially by SAAS to a designated adult Major Trauma Centre (Table 4). While the literature is conflicting regarding mortality outcomes for paediatric trauma patients managed in adult centres,^31^ initial transport to the paediatric MTC for these patients may have avoided these errors and would have avoided the need for secondary transfer.

This was a retrospective study and audit of practice initiated after a known adverse outcome in a patient where there was failure of TTA. Audits provide opportunities for systemic improvements and education, to ultimately improve patient care and patient outcomes.^32^ This retrospective study has all the standard limitations including incomplete information recorded in the medical record. At our institution, to meet reporting responsibilities to the Australian Trauma Registry, patients meeting TTA criteria are identified by Trauma Nurse Consultant review of the daily emergency department list and electronic medical record for all ED and PICU/HDU presentations and it is possible that some patients who met TTA criteria failed to be identified.

## Conclusion

The incidence of failure to initiate TTA in our paediatric major trauma centre was less than 5% and this suggests that our TTA criteria and trauma system are reasonably robust. Identification of factors associated with no TTA, such as direct PICU admissions, inter‐hospital transfers, injuries of regions other than limbs, younger patients and NAI, provides an opportunity to educate and prospectively audit to minimize future missed TTA. Other institutions may wish to review their own TTA performance to ensure they are not denying the spectrum of trauma system care to certain subsets of patients. The failure to initiate TTA for the patient whose death led to this audit serves as a tragic reminder of the important role TTA plays to optimize care for these vulnerable trauma patients.

## AUTHOR CONTRIBUTIONS

**Jonghoo Sung:** Conceptualization; methodology. **Angela Yao:** Conceptualization; methodology; supervision. **Georgia Antoniou:** Conceptualization; methodology; supervision. **Rebecca Cooksey:** Conceptualization; methodology; supervision. **Jacqueline Winters:** Conceptualization. **Michael Ee:** Conceptualization; investigation; methodology; supervision; validation. **Nicole Williams:** Conceptualization; investigation; methodology; supervision; validation.

## Conflict of interest

None declared.

## Data Availability

For queries regarding datasets generated during and/or analysed during the current study, please contact the corresponding author.
